# Improving fluid intelligence of children through working memory training: The role of inhibition control

**DOI:** 10.3389/fpsyg.2022.1025036

**Published:** 2022-11-25

**Authors:** Lei Wang, Ang Sheng, Lei Chang, Renlai Zhou

**Affiliations:** ^1^Department of Psychology, Nanjing University, Nanjing, Jiangsu, China; ^2^Department of Psychology, Faculty of Social Sciences, University of Macau, Taipa, Macao SAR, China; ^3^State Key Laboratory of Media Convergence Production Technology and Systems, Beijing, China

**Keywords:** fluid intelligence, working memory, inhibition control, N2, P3

## Abstract

Intelligence is strongly associated with working memory. Working memory training can improve fluid intelligence, but the underlying mechanism requires further investigation. Because inhibition control may play a key role in working memory training, this study investigated this process from an electrophysiological perspective. In total, 40 children aged 9 to 11 years were enrolled and randomly divided into a training group (n = 20) and a control group (n = 20). The training group received 20 days of working memory training, whereas the control group did not receive any training. Before and after the training period, all participants were tested using Raven’s Standard Progressive Matrices (RSPM), and electrophysiological indicators were recorded while they performed go/no-go and Stroop tasks. The results revealed that relative to the control group, the training group had significantly improved RSPM scores in the test conducted after their training. For the go/no-go tasks, the training group exhibited a significant decrease in N2 amplitude, a significant increase in P3 amplitude, a significant decrease in theta band energy, and an improvement in response inhibition ability. No significant change was observed for the Stroop task. Correlation analysis revealed that an improvement in individual response inhibition can positively predict an improvement in fluid intelligence. These results suggest that working memory training enhances the fluid intelligence of children by enhancing their response inhibition ability.

## Introduction

Intelligence comprises two components, namely fluid intelligence, which is closely related to problem solving and abstract reasoning and crystalized intelligence, which pertains to knowledge accumulation (Gray et al., 2003; Gray and Thompson, 2004). Studies have indicated that the ability of an individual to engage in abstract reasoning, problem solving, and fast learning is highly dependent on their working memory ability (Colom et al., 2004; Oberauer et al., 2005). Some researchers have argued that the function and structure of working memory are the basis of fluid intelligence (Oberauer et al., 2007).

Studies have discovered that the effects of working memory training can be transferred to fluid intelligence (Jaeggi et al., 2008). In a study by Zhao et al. (2011), 16 children aged 9–11 years received working memory training for 15 days, after which their fluid intelligence was improved. The effect of working memory training has also been verified in children with cognitive deficits. This form of training not only improves cognitive deficits associated with learning disabilities but also improves the working memory, fluid intelligence, and math performance of children with learning disabilities (Chen et al., 2017).

Although scholars have verified that working memory training has a far-transfer effect on the fluid intelligence of children, the related neural mechanisms are not well understood. Chein and Morrison (2010) argued that working memory training transfers the training effect to fluid intelligence by improving inhibition control (Kane et al., 2001; Engle, 2002; Klingberg et al., 2002; Klingberg, 2010; Au et al., 2015; Greenwood and Parasuraman, 2015; Au et al., 2016; Ye et al., 2018), which is a core component of the executive function of working memory. Furthermore, inhibition control is a top-down ability that enables an individual to actively interrupt or delay their behavior (Clark, 1996; DeWall et al., 2011; Brydges et al., 2012; Diamond, 2013) and engage in the purposeful detection and monitoring of target-oriented behaviors (Cattell, 1963; Kane and Engle, 2002; Rueda, 2018; Rico-Picó et al., 2021). Children and adults with higher fluid intelligence exhibit higher inhibition control and efficiency (Burgess et al., 2011); this suggests that inhibition control is a core component of fluid intelligence, possibly because of their shared neural mechanisms (Duncan and Owen, 2000; Cowan et al., 2006; Jung and Haier, 2007). Fjell et al. (2015) examined the development of the cerebral cortex and fluid intelligence and reported a significant positive correlation between fluid intelligence and prefrontal lobe development, especially the development of the anterior cingulate cortex (ACC). The ACC is a key node in the inhibition control network and plays a crucial role in the development of fluid intelligence.

Demetriou et al. (2008) proposed a hierarchical model of intelligence in which fluid intelligence (with reasoning as its core) is based on inhibition control, processing efficiency, and other related low-level cognitive processes. Studies have demonstrated that children with higher fluid intelligence can inhibit interference more effectively in tests, and on this basis, they can employ cognitive strategies to create, update, maintain, and manipulate abstract representations to achieve better fluid intelligence outcomes (Rueda, 2018). Wiley et al. (2011) further linked this process to working memory, arguing that inhibition control can explain why people with superior working memory perform better on Raven’s Standard Progressive Matrices (RSPM; Raven, 2000). When individuals must adhere to new rules in problem-solving situations, the rules that they previously learned and applied may affect their problem-solving efficiency. Compared with individuals with superior working memory, those with poorer working memory find it more difficult to refocus away from the previously learned rules and they persist in applying these outdated rules to solve new problems. Their problem-solving ability is thus negatively affected. Gray et al. (2003) revealed that for high-load updating tasks, participants with higher fluid intelligence had a higher task accuracy rate. The results of multiple regression analysis suggested that the lateral prefrontal cortex and frontoparietal lobe may mediate the relationship between fluid intelligence and working memory. The discussed results suggest that inhibition control plays an essential role in the connection between working memory and fluid intelligence. However, few studies have used event-related potential to explore the change in inhibition control during the transfer of working memory training effects to fluid intelligence.

Researchers have categorized inhibition control into interference inhibition and response inhibition (Nigg, 2000; Johnstone et al., 2009; Diamond, 2013). Interference inhibition (also referred to as conflict resolution and executive attention) is the ability of individuals to focus their attention on the current task through willful effort, with the aim of excluding or inhibiting interfering information that is unrelated to the task. Conversely, response inhibition refers to the ability of individuals to inhibit behavioral responses that do not meet their current needs (Johnstone et al., 2009; Diamond, 2013). Various theories for explaining the inhibition control mechanism have been proposed, and the major ones are feature integration theory (Nieuwenhuis et al., 2003; Hommel et al., 2004; Fischer et al., 2010; Ye et al., 2019) and conflict monitoring theory (Botvinick et al., 2001; Milham et al., 2003; Carter and van Veen, 2007; Clayson and Larson, 2011). Under feature integration theory, the inhibition control process is automatic. An individual reacts accordingly when they encounter a stimulus. At this point, the brain integrates and stores stimulus and response features. When the individual is executing subsequent tasks, the reappearance of these stimulus features automatically triggers the response that is associated with them, thereby reducing the individual’s response time and producing an adaptation effect. However, when the individual encounters a completely new stimulus feature, the feature conflicts with their stored integration mode such that a longer response time is required. Under conflict monitoring theory, a cognitive system is required to exert active control in the process of inhibition control. During the early stage of inhibition control, the ACC is activated when interference information appears, and this process plays a role in conflict monitoring and exploration. When conflict is detected, conflict signals from the ACC increase the activity in the prefrontal cortex, which, in turn, enhances top-down cognitive control. Although interference control and response inhibition are collectively referred to as inhibition control, these two inhibition processes exhibit different patterns in terms of their activation of inhibition behavior, namely top-down cognitive drive and bottom-top stimulus drive. Hong et al. (2017) manipulated go/no-go tasks, which pertain to response inhibition, and their results suggested that an individual’s top-down cognitive drive is a fundamental prerequisite for such inhibition. Interference control that inhibits task-irrelevant information is regarded as being determined by bottom-top stimulation processing. The features of an interfering stimulus automatically activate the cognitive representation of the relevant response of the individual that would occupy cognitive resources and thus create interference for the individual (Avital-Cohen and Tsal, 2016). Therefore, the present study used go/no-go and Stroop tasks to comprehensively explore the role of inhibition control in the transfer of working memory training effects to fluid intelligence.

The N2 is an event-related potential (ERP) indicator of response inhibition control or conflict monitoring. During the execution of Stroop and go/no-go tasks, N2 components appear after a stimulus triggers inhibitory control (Folstein and Petten, 2008). Source localization findings suggest that N2 is derived from activation of the ACC, reflecting the top-down monitoring of conflict and amount of effort required to complete an inhibition control task (Nieuwenhuis et al., 2003; Pires et al., 2014). Generally, larger N2 amplitude indicates superior conflict monitoring capability. However, developmental studies have reported that N2 amplitude decreases with age, which can be interpreted as an increase in conflict monitoring efficiency (Lo, 2018). P3 components are related to the inhibition control process, and studies have reported that individuals with high fluid intelligence exhibited higher P3 amplitude after completing an inhibition control task, which could reflect a greater degree of mature inhibition control (Wessel, 2018; Rico-Picó et al., 2021).

The ERP index only indicates that a brain is time- and phase-locked to the onset of a stimulus or a response. However, the electrophysiological activity of a brain that is time-locked but not phase-locked to the onset of a stimulus or response (i.e., event-related spectral perturbation [ERSP]) may reveal novel neural mechanisms that are involved in conflict detection, monitoring, and resolution (Makeig et al., 2004). A study demonstrated that inhibition control tasks are accompanied by theta (4–8 Hz) and alpha (8–13 Hz) energy changes. An increase in theta band energy in the prefrontal lobe may reflect top-down inhibition control of a resource input during the processes of individual identification, monitoring, and problem solving (Nigbur et al., 2012; PastöTter et al., 2013; Cavanagh and Frank, 2014). An intensely debated topic is the change in the alpha band energy in the parietal lobe, which may reflect the investment of inhibition control resources and reinforcement of inhibition control by a task-independent brain (Pfurtscheller et al., 1996; Klimesch et al., 2007; Klimesch, 2012).

The present study primarily aimed to explore the role of inhibition control during the transfer of working memory training effects to fluid intelligence in children from the perspective of electrophysiology. Therefore, we compared the electrophysiological performance of two groups of children with respect to the execution of inhibition control tasks. Studies have indicated that the gradual transition from bottom-top stimulation drive to top-down cognitive control is the basis for enhancing the mental representation of children. This transition ability enables children to better inhibit previously applied rules during fluid intelligence tasks, focus their attention on cues that are relevant to the current task, and respond flexibly to new situations (Munakata et al., 2012). We hypothesized that the significant improvement in the fluid intelligence of the children in the training group was influenced by the improvement in top-down internally driven response inhibition that was achieved through working memory training; by contrast, no significant change in bottom-top stimulus-driven interference inhibition was detected during this process. In other words, the N2 amplitude significantly decreased, P3 amplitude significantly increased, theta energy level increased, and alpha energy level decreased during the execution of go/no-go tasks. No significant change was detected for Stroop task indices.

## Materials and methods

### Participants

The desired sample size was calculated by performing a G*Power analysis. With *f* = 0.25, *α* = 0.05, and power = 0.8, we obtained a recommended sample size of 34 participants (Faul et al., 2007). In total, 40 children (aged between 10 and 11 years) from a primary school in Nanjing were enrolled and randomly divided into two groups with 20 children each, namely the control group (mean age, 10.35 ± 0.64 years; 10 boys and 10 girls) and training group (mean age, 10.36 ± 0.49 years; 12 boys and 8 girls). No significant difference in age was observed between the two groups (*t* = 0.058, *p =* 0.954). None of the participants had previously participated in a similar study. The experimental procedures were approved by the Ethics Committee of the Department of Psychology, Nanjing University, and they were performed in accordance with approved guidelines. In accordance with the Declaration of Helsinki, informed consent was obtained from the parents and teachers of the participants.

### Materials and procedure

#### Training tasks

**Figure 1:**
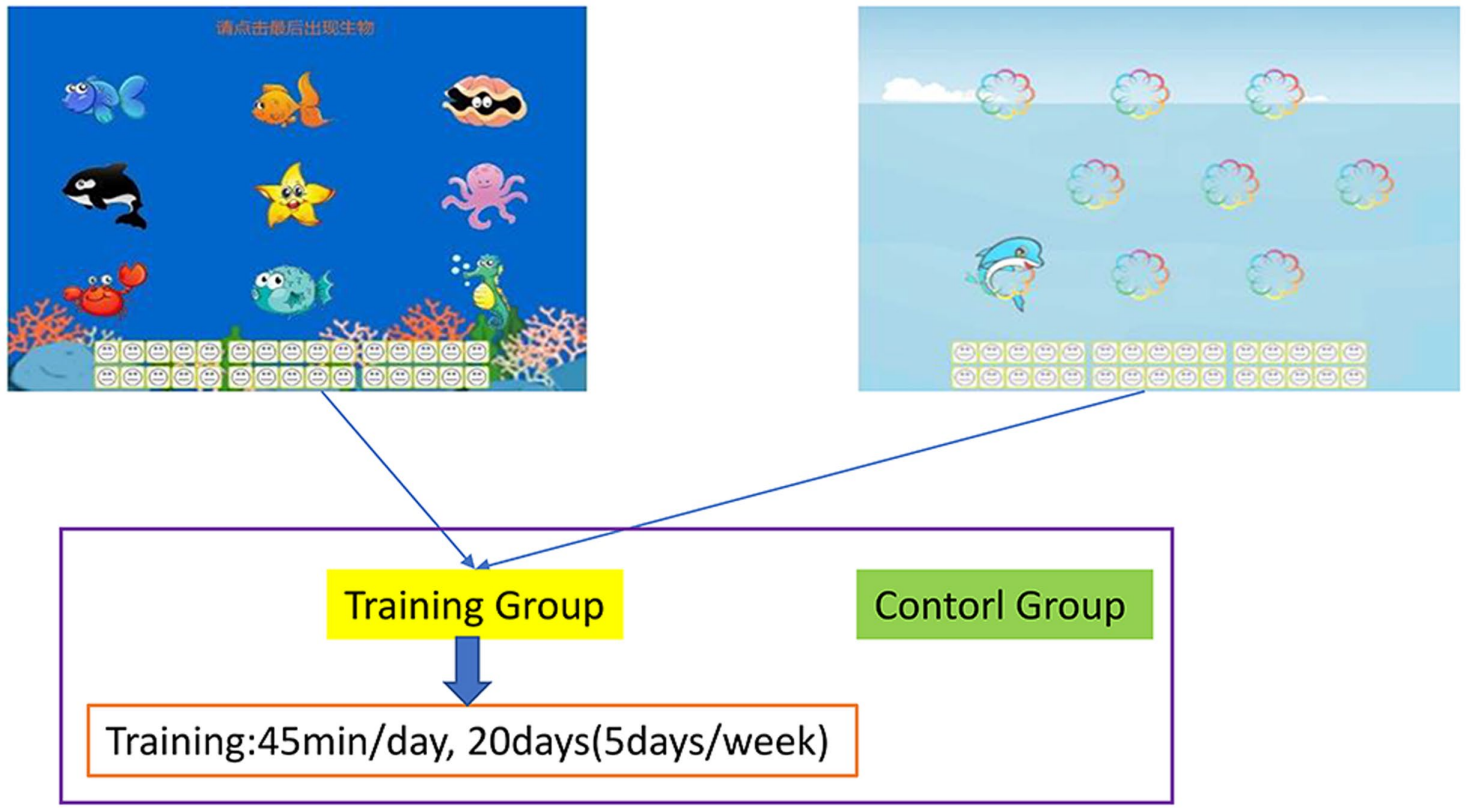
Working memory training task. Participants were required to memorize the final three images that were presented. The training task was divided into six blocks of five trials. When a participant’s correct response rate increased, their stimulus presentation time was reduced by 100 ms for the subsequent block, which increased the difficulty of the task.

In the present study, an original training program was optimized to develop a new version of adaptive working memory training task software (Zhao et al., 2011). During training tasks, various animals, numbers, and robots were presented sequentially in the center of a computer screen. The participants did not know the number of animals, numbers, and robots that would appear during each trial and were instructed to memorize the identity of the final three animals or numbers that appeared during each trial. They had to continually update their memory and thus train their working memory because they did not know how many animals, numbers, and robots would be presented. Each training task comprised 30 trials that were separated into six blocks of 5 trials. At the beginning of training, each item was presented for 1.750 ms. This duration was reduced by 100 ms for the subsequent block if a participant provided correct responses for three or more trials in a given block. The duration of training that a participant received on a subsequent day was determined by the duration of the participant’s last block on a given day. The participants received feedback on their overall performance (Figure 1).

#### Evaluation of training outcomes

##### RSPM

An RSPM test was performed to measure the fluid intelligence of the participants. RSPM is a reasoning problem that comprises abstract geometric figures or line segments. This test is divided into five components (A, B, C, D, and E) that each comprise 12 questions (i.e., the test has 60 questions in total). Each correct answer was assigned 1 point. An improvement in RSPM score is analogous to an improvement in fluid intelligence. In accordance with the protocol used by Jaeggi et al. (2008), we divided the test into two equal parts on the basis of pen-and-paper administration. All participants completed the test in a school classroom under the guidance and supervision of professional trainers. On average, the participants required 20 min or less to complete 30 questions.

##### Stroop task

**Figure 2:**
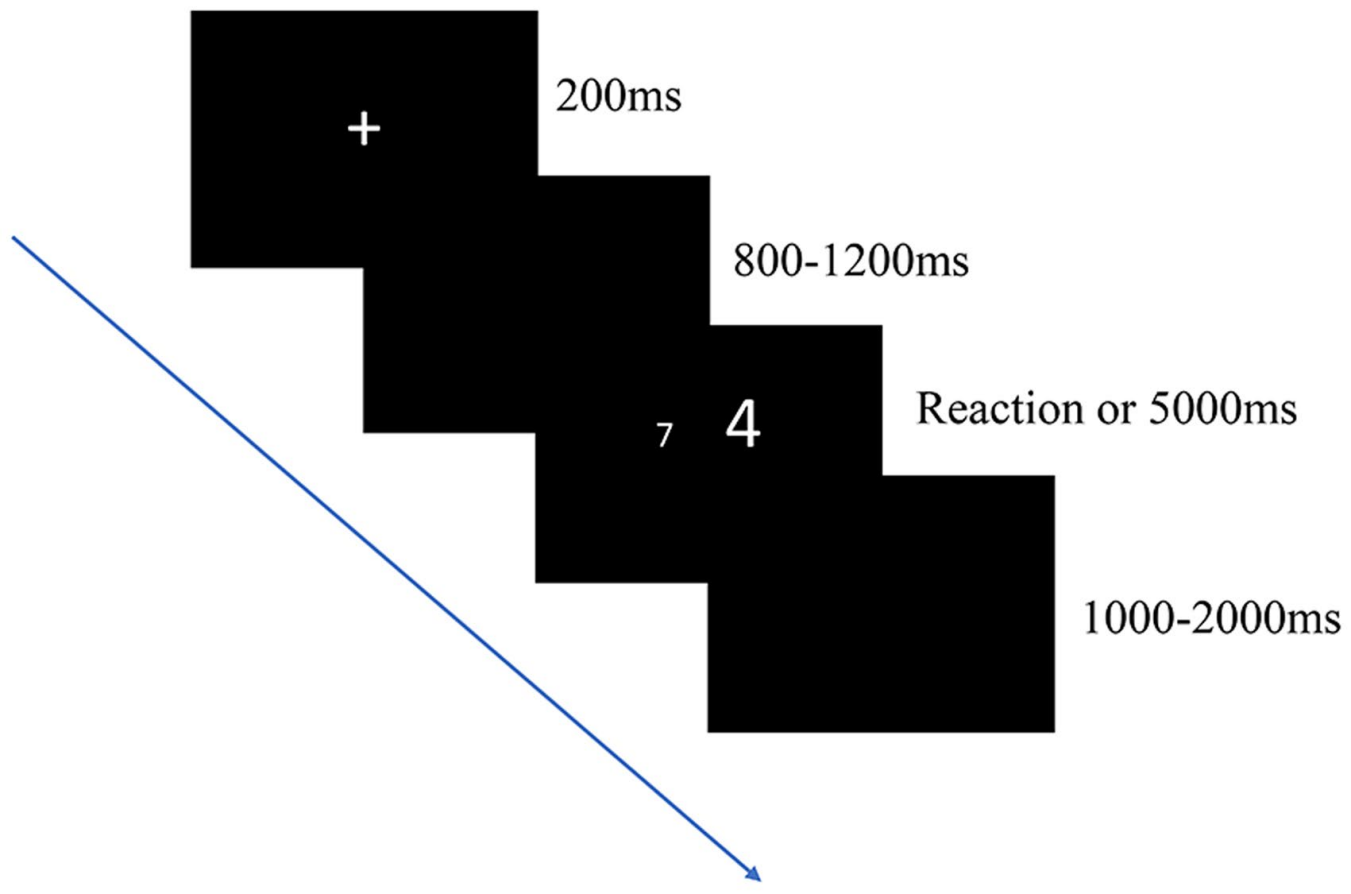
Digital Stroop task. The participants judged the numerical value of the number shown on the screen while ignoring its physical size. Three conditions were applied (incongruent, congruent, and neutral conditions), with the probability of each condition occurring being 0.33.

In the present study, the Stroop task was used to evaluate the conflict inhibition of the participants (Liu et al., 2011). The experimental program was developed using the E-prime software, and its screen background and stimulus material were black and white, respectively. The distance between a participant and the screen was 70–100 cm. Each participant was asked to compare the numerical values of two white single-digit numbers against a gray background. To reduce the effect of distance, one digit in each number pair was programmed to be greater than the other by a value of 3 (i.e., 1–4, 2–5, 3–6, 4–7, 5–8, and 6–9). The numerically greater digit was presented randomly. The participants were exposed to one of three conditions, namely the congruent, incongruent, and neutral conditions. Each condition was tested twice (pretraining and posttraining). Under the congruent condition, the numerically greater digit (200 points) was physically larger than the other (140 points). Under the incongruent condition, the numerically greater digit was physically smaller than the other. Under the neutral condition, both digits had physically identical size (half of 140 points and half of 200 points). During execution of a Stroop task, each trial began with a fixation cross “+” for 200 ms, which was displayed randomly on the screen for 800–1,200 ms after stimulus presentation. The subjects were asked to judge which number was large (i. e. to judge which number has a large value, ignoring the physical size of the number), if the large number is on the left, press the “1” key on the digital keypad; if the large number is on the right, press the “3” key on the digital keypad. The stimulus remained for a period of time (5,000 ms) or until a response was given. The next trial was introduced after 1,000 to 2,000 ms, that is, the intertrial intervals varied between 1,000 and 2,000 ms. The timing of the trial times under consistent, inconsistent, and neutral conditions was randomized, but the same key was not allowed to appear for more than four consecutive times, and the same condition was not allowed to be applied for more than three consecutive times (Figure 2).

##### Go/no-go task

**Figure 3:**
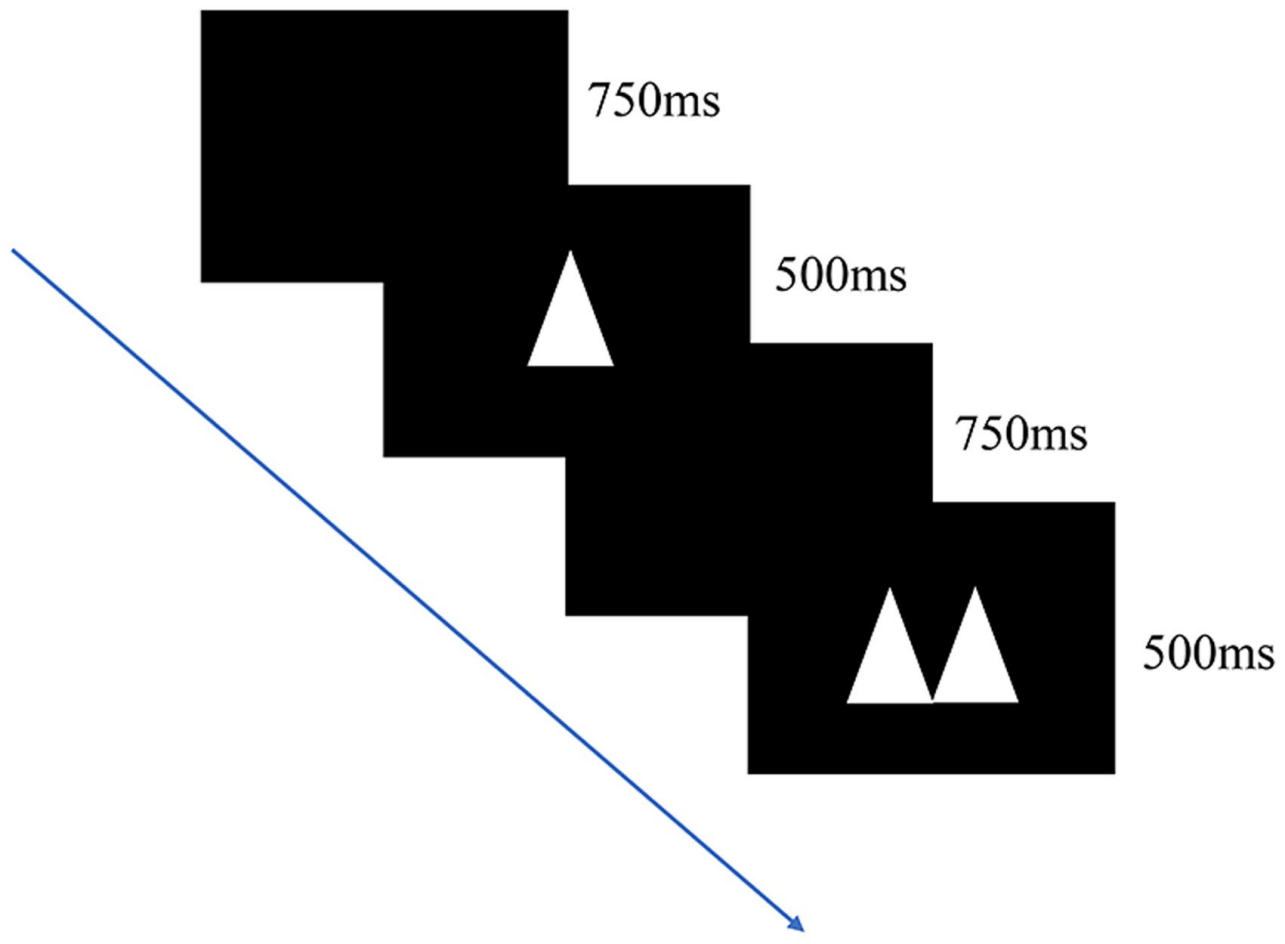
Digital go/no-go task. The participants responded to images displayed on screen by pressing “/” button when double triangles appeared but not when a single triangle appeared. Each trial had a 60% chance of being a go trial and 40% chance of being a no-go trial.

The response inhibition ability of the participants was measured using the go/no-go task. The task requires a participant to react when they observe a target stimulus but not when they observe a nontarget stimulus. The stimulus material comprised double and single triangles, and the order in which double and single triangles were presented was fully randomized. The task consisted of a practice block and three formal experimental blocks. During the formal experiment, the ratio of go trials to no-go trials was 60%:40%. The participants were required to press the “/” button when they observed double triangles (go trial) and to not press any button when they observed a single triangle (“no-go” trial). Each block comprised 100 trials for a total of 300 trials. During each trial, a triangle was randomly presented in the center of the computer screen for 500 ms, after which a blank screen was displayed for 750 ms prior to the start of the subsequent trial (Figure 3).

### Design and procedure

The working memory of the experimental group was trained. The training consisted of 20 sessions. Each session lasted between 15 and 20 min, and between three and four training sessions were conducted on a weekly basis. The training program was conducted using adaptive working memory training software developed on the basis of the findings of Zhao et al. (2011). The participants completed the computer-based training during the daily noon break (12:00 to 1:30 p.m.) in their school’s computer room under the guidance and supervision of professional trainers. The experiment comprised two components, namely the pretraining and posttraining tests (test sequence: rspm, stroop, and go/no-go). The posttraining test was conducted after the experimental group had completed the 20 training sessions. The experimental group received the training and completed all of the tests, whereas the control group did not receive any specific training and only completed the tests. Both groups completed the same tasks and tests.

### Electroencephalography data collection and analysis

We recorded EEG data while the participants completed the Stroop and go/no-go tasks. A Neuroscan 40-channel portable amplifier was used to record EEG data through DC sampling. Electrodes were arranged on a cap in accordance with the standard international 10–20 system. The reference electrodes were attached to the mastoids in the left ear, and the obtained data were converted into a mean reference for bilateral mastoids during our offline analysis. The sampling rate was 1,000 Hz, and the recording bandwidth was 0.01–100 Hz. The ground electrode was attached to the midpoint AFz of the FCz and Fz connection. HEOG data were recorded by electrodes placed on the lateral side of both eyes, and VEOG data were recorded by electrodes placed on the above and below sides of the left eye. Reference electrodes were attached to the left mastoid process, and the input impedance of all recording electrodes was less than 10 kΩ. Because the acquisition environment was not electromagnetically shielded, a 50-Hz notch filter was used to subtract urban electrical interference.

EEG data were processed using the EEGLAB 2019 toolkit (Delorme and Makeig, 2004), which is based on the MATLAB2019b platform. Continuous EEG data were filtered using a 40-Hz low-pass filter and a 0.1-Hz high-pass filter and subsequently re-referenced to an average signal. The data were segmented with a time window of 900 ms (100 ms before stimulation and 800 ms after stimulation) and a prestimulus baseline of 100 ms. Trials with considerable drift were manually removed, and those that were contaminated by eye blinks were corrected using an independent component analysis algorithm (infomax; Delorme and Makeig, 2004). Across the participants, 3 ± 2 independent components of artifacts were identified as ocular artifacts through visual inspection and were rejected. Only correct trials were included in the final analysis. Finally, the trials in which amplitude values exceeding ±75 μV were obtained for any electrode were excluded from the analysis.

The channels and time windows for the analyses were selected on the basis of the literature regarding developmental samples (Wessel, 2018; Rico-Picó et al., 2021; Fu et al., 2022). Imperative No-go stimuli typically produce a negative deflection in the ERP around 200–300 milliseconds, the N2, that is maximal over fronto-central regions (Van Veen and Carter, 2002). For the N2 component, Fz was selected for analysis. The average amplitude of the EEG data within the 290–360 ms time window after stimulation served as the N2 for analyzing the Go/no-go, and the average amplitude of the EEG data within the 280–350 ms time window after stimulation served as the N2 for analyzing the Stroop task. The channels Fz and Cz were used to examine P3. For both tasks, the selected time window for P3 was between 350 and 500 ms after the stimulation.

For the time–frequency analysis, Letswave7, which is based on the MATLAB 2019b platform, was used to perform a continuous Morlet wavelet transform of the data, and oscillation power was estimated using single-trial EEG epochs. To simultaneously obtain favorable temporal and spatial resolutions, a frequency range of 1–30 Hz and data in the range of −600 to 800 ms were selected for segmentation in steps of 10 ms. To prevent edge effects during a continuous Morlet wavelet transform, the prestimulus time interval (−400 to −200 ms) was used as the baseline interval. For the go/no-go and Stroop tasks, channels were selected on the basis of the findings of other studies (Klimesch, 2012; Cavanagh and Frank, 2014). Specifically, the channel Fz was selected for the analysis of theta band energy for the Stroop task (frequency range, 4–8 Hz; time window, 150–350 ms), and the channel Cz was selected for the analysis of alpha band energy (frequency range, 8–13 Hz; time window, 400–650 ms).

## Results

### RSPM

**Figure 4:**
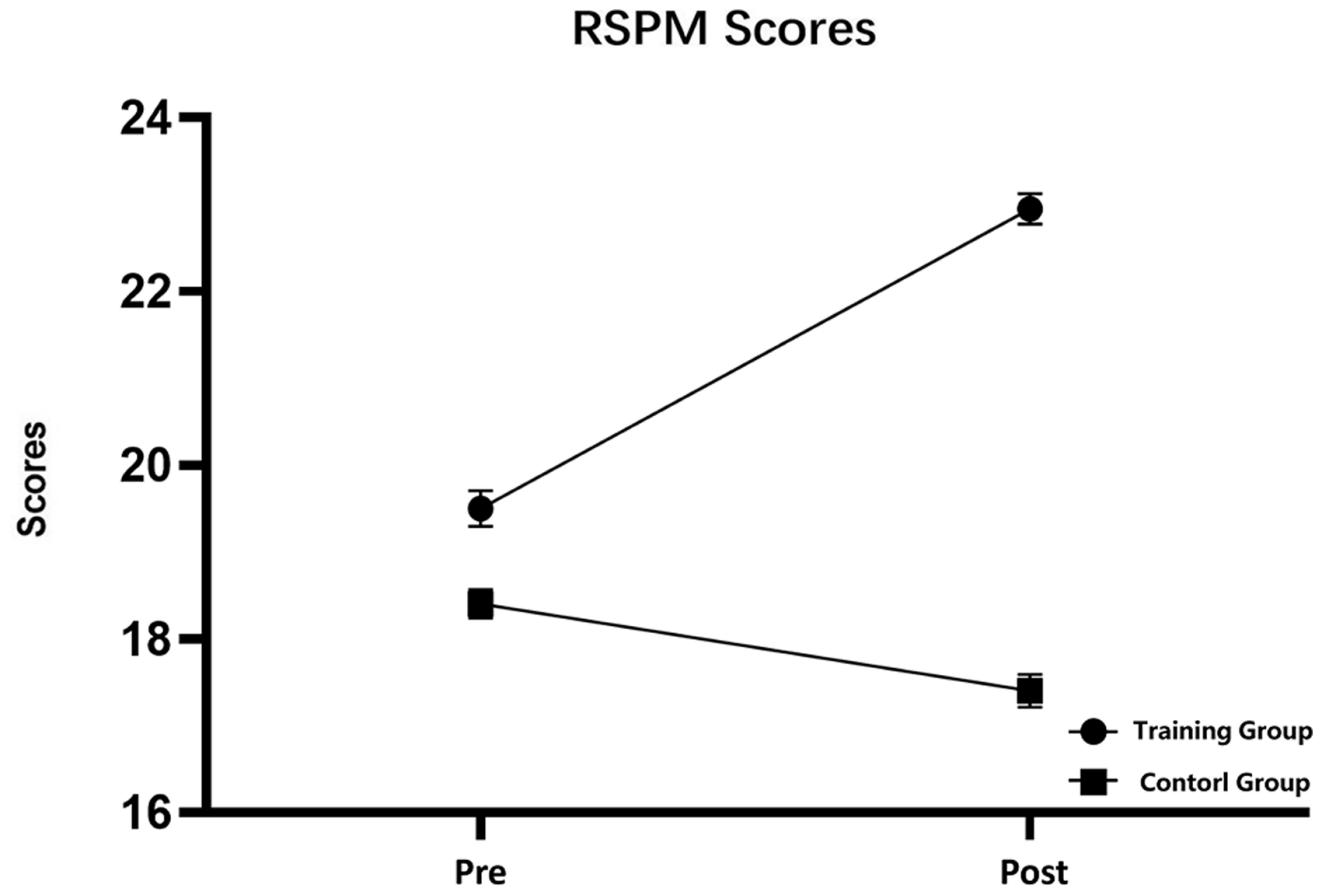
Raven’s Standard Progressive Matrices scores of the participants in the pretest and posttest. The training group had significantly improved RSPM scores after receiving working memory training. No significant difference in scores was detected in the control group. Pre, training group pretest and Control-pre; Post, training group posttest and Control-post, error bars are standard error.

RSPM scores were subjected to 2 (training group and control group) × 2 (pretest and posttest) repeated-measures analysis of variance (RM-ANOVA). The RM-ANOVA revealed the following results. The main effect of time was nonsignificant (*F* (1, 38) = 2.274, *p* = 0.140, *η^2^* = 0.056), the main effect of group was significant (*F* (1, 38) = 13.909, *p* = 0.001, *η^2^* = 0.268), and the interaction between time and group was significant (*F* (1, 38) = 7.503, *p* = 0.009, *η^2^* = 0.165). Simple effect analysis revealed that the RSPM scores of the training group were significantly enhanced after the training (*F* (1, 38) = 9.019, *p* = 0.005, *η^2^* = 0.192), whereas those of the control group did not differ significantly between the start and end of the present study (*F* (1, 38) = 0.758, *p* = 0.389, *η^2^* = 0.02) (Figure 4).

### Go/no-go task

No-go trials tested the response inhibition of the participants. Thus, only the amplitude of the no-go responses of the participants was analyzed. This principle was applied as follows.

#### N2

**Figure 5:**
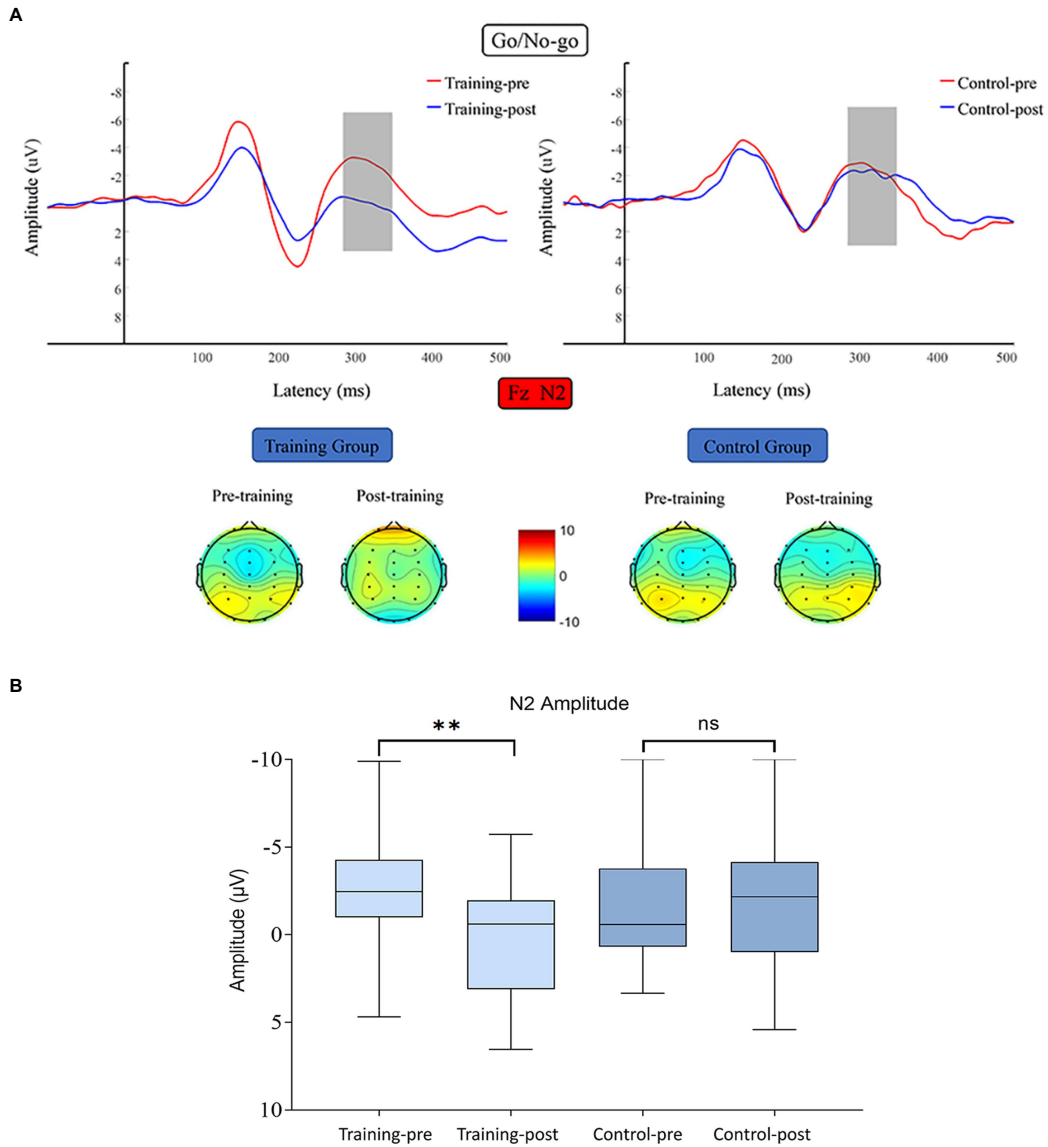
**(A)** Waveforms and topographical maps of N2 for the two groups. Topographical maps were constructed using the mean amplitude for the N2 period (290–360 ms). **(B)** Bar chart of the amplitude of N2 along the Fz channel between 290 and 360 ms. Bars represent the confidence interval. *6 edges **p < 0.01, *ns* indicates *p >* 0.05. Training-pre, training group pretest; Training-post, training group posttest; Control-pre, control group pretest; Control-post, control group posttest.

N2 average amplitude data were subjected to 2 (training group and control group) × 2 (pretest and posttest) RM- ANOVA, which revealed the following results. The main effect of time was significant (*F* (1, 38) = 4.811, *p* = 0.034, *η^2^* = 0.112), the main effect of group was nonsignificant (*F* (1, 38) = 0.627, *p* = 0.433, *η^2^* = 0.016), and the interaction between time and group was significant (*F* (1, 38) = 4.272, *p* = 0.046, *η^2^* = 0.101). Simple effect analysis indicated that the N2 amplitude of the training group was significantly decreased after the training, *F* (1, 38) = 9.075 *p* = 0.005, *η^2^* = 0.193). By contrast, that of the control group did not differ significantly between the start and end of the present study (*F* (1, 38) = 0.008, *p* = 0.929, *η^2^* < 0.001; Figure 5).

#### P3

**Figure 6:**
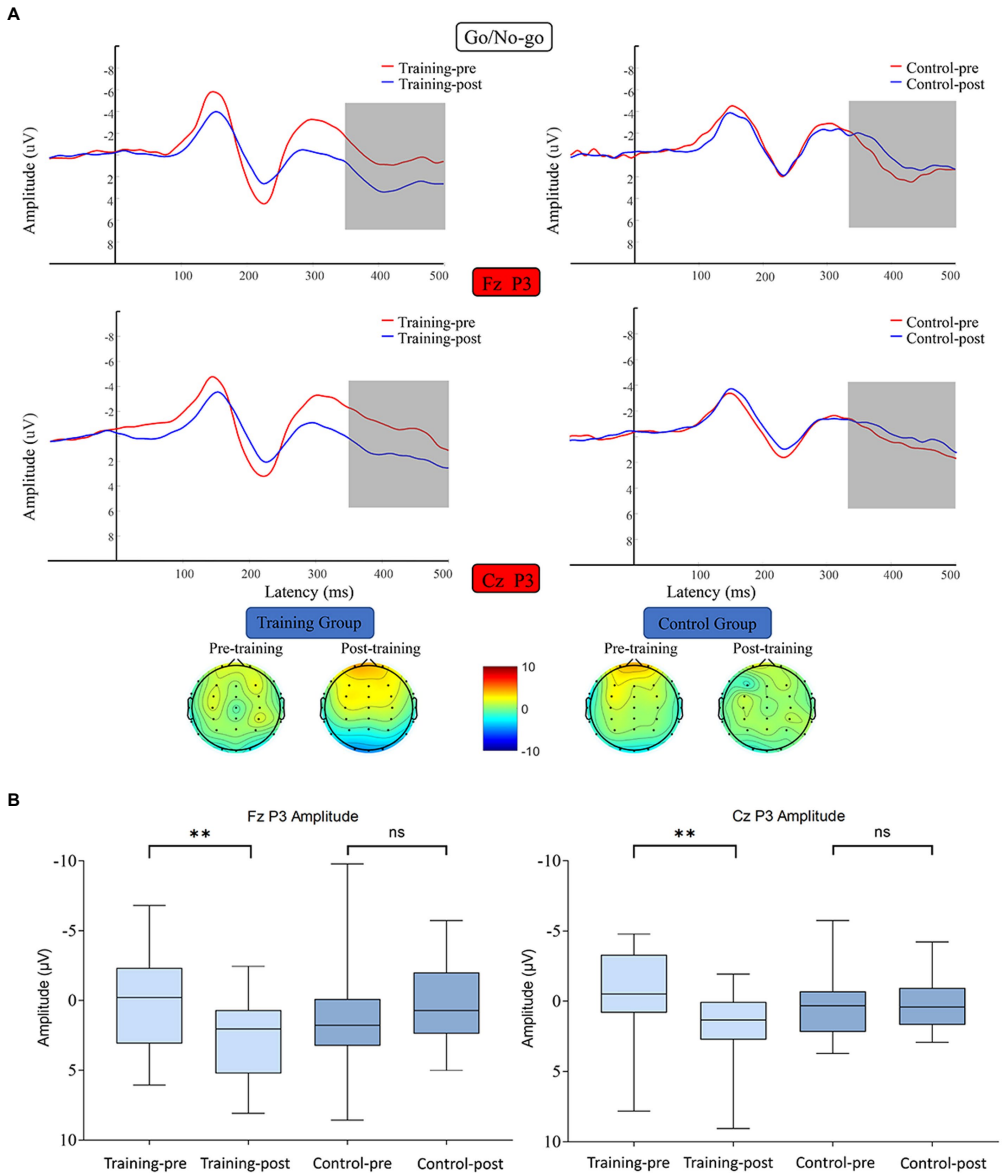
**(A)** Waveforms and topographical maps of P3 for the two groups along Fz and Cz. Topographical maps were constructed using the mean amplitude for the P3 period (350–500 ms). **(B)** Bar chart of the amplitude of P3 along the Fz and Cz channels between 350 and 500 ms. Bars represent the confidence interval. *6 edges **p < 0.01, *ns* indicates *p >* 0.05. Training-pre, training group pretest; Training-post, training group posttest; Control-pre, control group pretest; Control-post, control group posttest.

P3 average amplitude data were subjected to 2 (training group and control group) × 2 (pretest and posttest) × 2 (Fz and Cz) RM-ANOVA. The main effect of time was significant (*F* (1, 38) = 5.654, *p* = 0.023, *η^2^* = 0.130), and the interaction between time and channels was significant (*F* (1, 38) = 18.592, *p* < 0.001, *η^2^* = 0.329). The data on the mean amplitude of P3 along the channels Fz and Cz were subjected to 2 (training group and control group) × 2 (pretest and posttest) RM-ANOVA. Regarding the Fz channel, the main effect of time was marginally unsignificant (*F* (1, 38) = 2.052, *p* = 0.160, *η^2^* = 0.051), and the interaction between time and group was significant (*F* (1, 38) = 13.539, *p* = 0.001, *η^2^* = 0.263). Simple effect analysis revealed that the P3 amplitude along the Fz of the training group was significantly enhanced after the training (*F* (1, 38) =13.066, *p* = 0.001, *η^2^* = 0.256), whereas that of the control group did not differ significantly between the start and end of the study (*F* (1, 38) = 2.525, *p* = 0.120, *η^2^* = 0.062). Regarding the Cz channel, the main effect of time was unsignificant (*F* (1, 38) = 2.890, *p* = 0.097, *η^2^* = 0.071), and the interaction between time and group was significant (*F* (1, 38) = 9.294, *p* = 0.004, *η^2^* = 0.197). Simple effect analysis indicated that the P3 amplitude along the Cz channel was significantly enhanced after the training in the training group (*F* (1, 38) = 11.274, *p* = 0.002, *η^2^* = 0.229), whereas in the control group, this amplitude did not differ significantly between the start and end of the study (*F* (1, 38) = 0.910, *p* = 0.346, *η^2^* = 0.023) (Figure 6).

#### Time–frequency analysis

**Figure 7:**
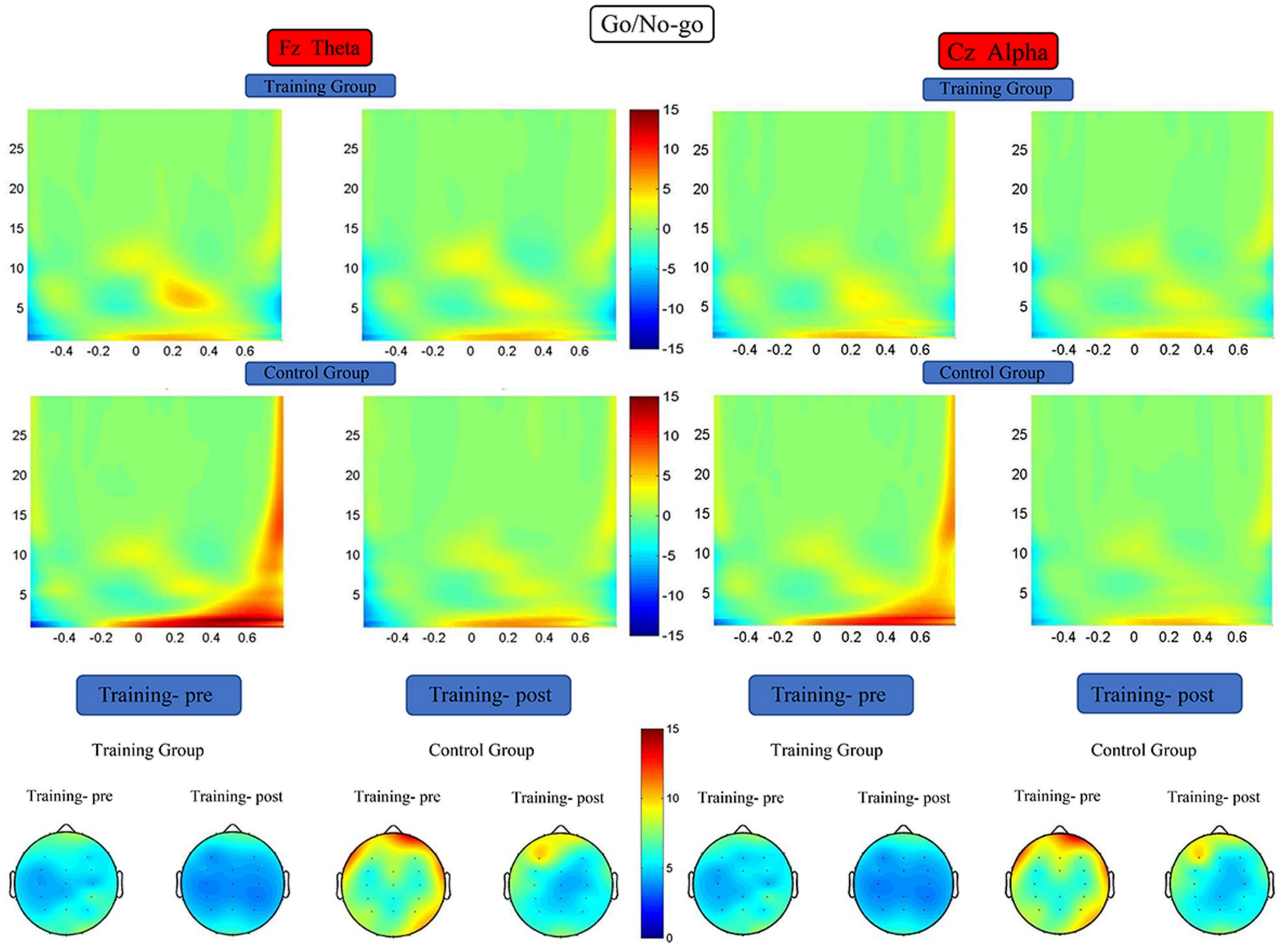
Spectrograms of mean ERSP of the two groups at electrodes Fz and Cz. Training-pre, training group pretest; Training-post, training group posttest; Control-pre, control group pretest; Control-post, control group posttest.

For the Fz channel, theta energy data were subjected to 2 (training group and control group) × 2 (pretest and posttest) RM-ANOVA, which revealed the following. The main effect of time was significant (*F* (1, 38) = 6.954, *p* = 0.012, *η*^2^ = 0.155), the main effect of group was significant (*F* (1, 38) = 5.228, *p* = 0.028, *η^2^* = 0.121), and the interaction between time and group was significant (*F* (1, 38) = 5.063, *p* = 0.030, *η*^2^ = 0.118). Simple effect analysis indicated that the theta energy level of the training group was significantly higher after the training (*F* (1, 38) = 11.942, *p* = 0.001, *η*^2^ = 0.239), whereas that of the control group did not differ significantly between the start and end of the study (*F* (1, 38) = 0.075, *p* = 0.786, *η*^2^ = 0.002). For the Cz channel, alpha energy data were subjected to 2 (training group and control group) × 2 (pretest and posttest) RM-ANOVA, which did not reveal any significant difference in the alpha energy levels of the two groups before versus after the training period (Figure 7).

### Stroop task

The amplitude under the incongruent condition within the Stroop task reflected the response inhibition of the participants. Thus, the amplitude obtained under only the incongruent condition was analyzed.

#### N2

**Figure 8:**
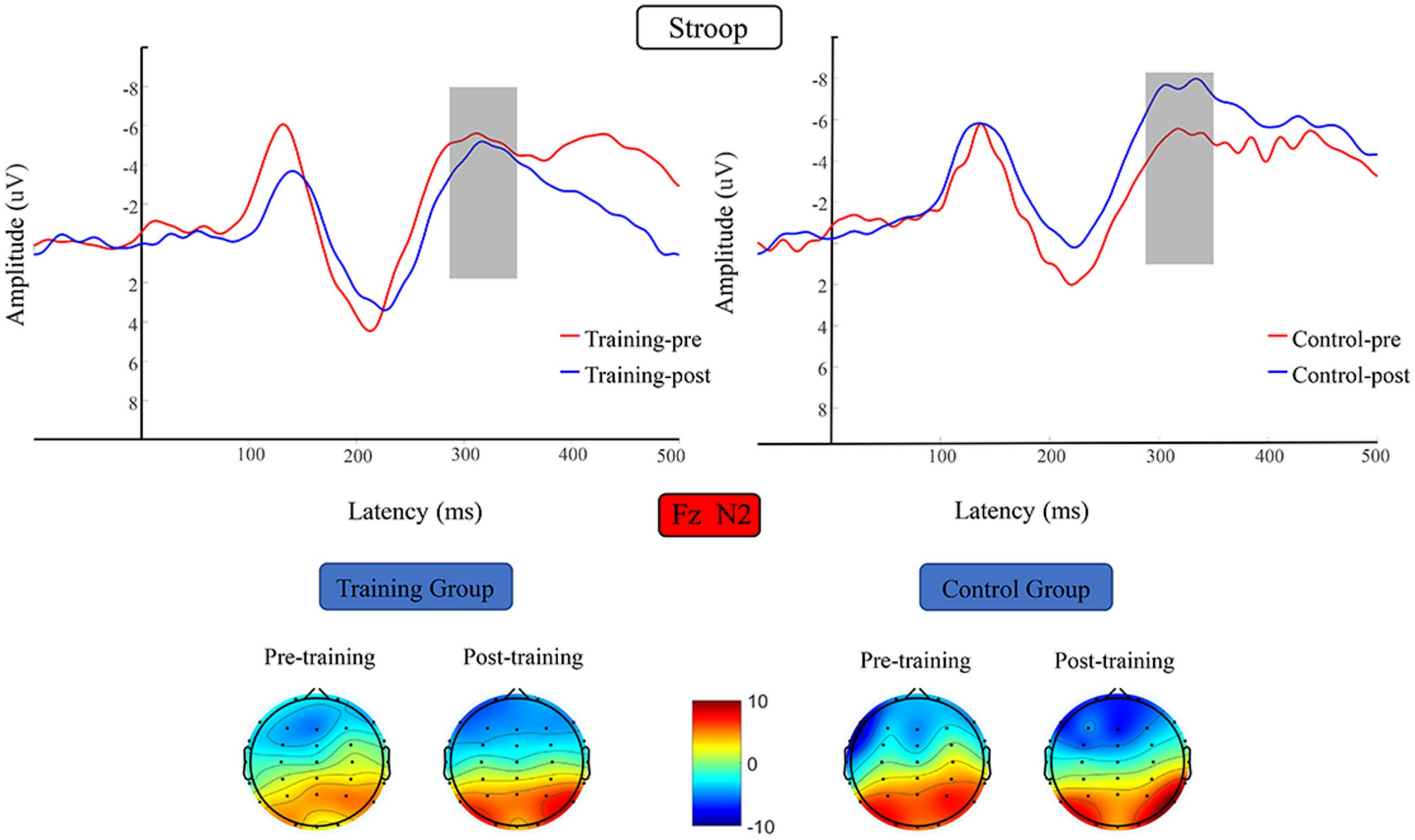
Waveforms and topographical maps of N2 for the two groups. Topographical maps were constructed using the mean amplitude in the N2 period (280–350 ms). Training-pre, training group pretest; Training-post, training group posttest; Control-pre, control group pretest; Control-post, control group posttest.

The N2 average amplitude data were subjected to 2 (training group and control group) × 2 (pretest and posttest) RM-ANOVA, which did not reveal any significant difference in N2 average amplitude before versus after the training period (Figure 8).

##### P3

**Figure 9:**
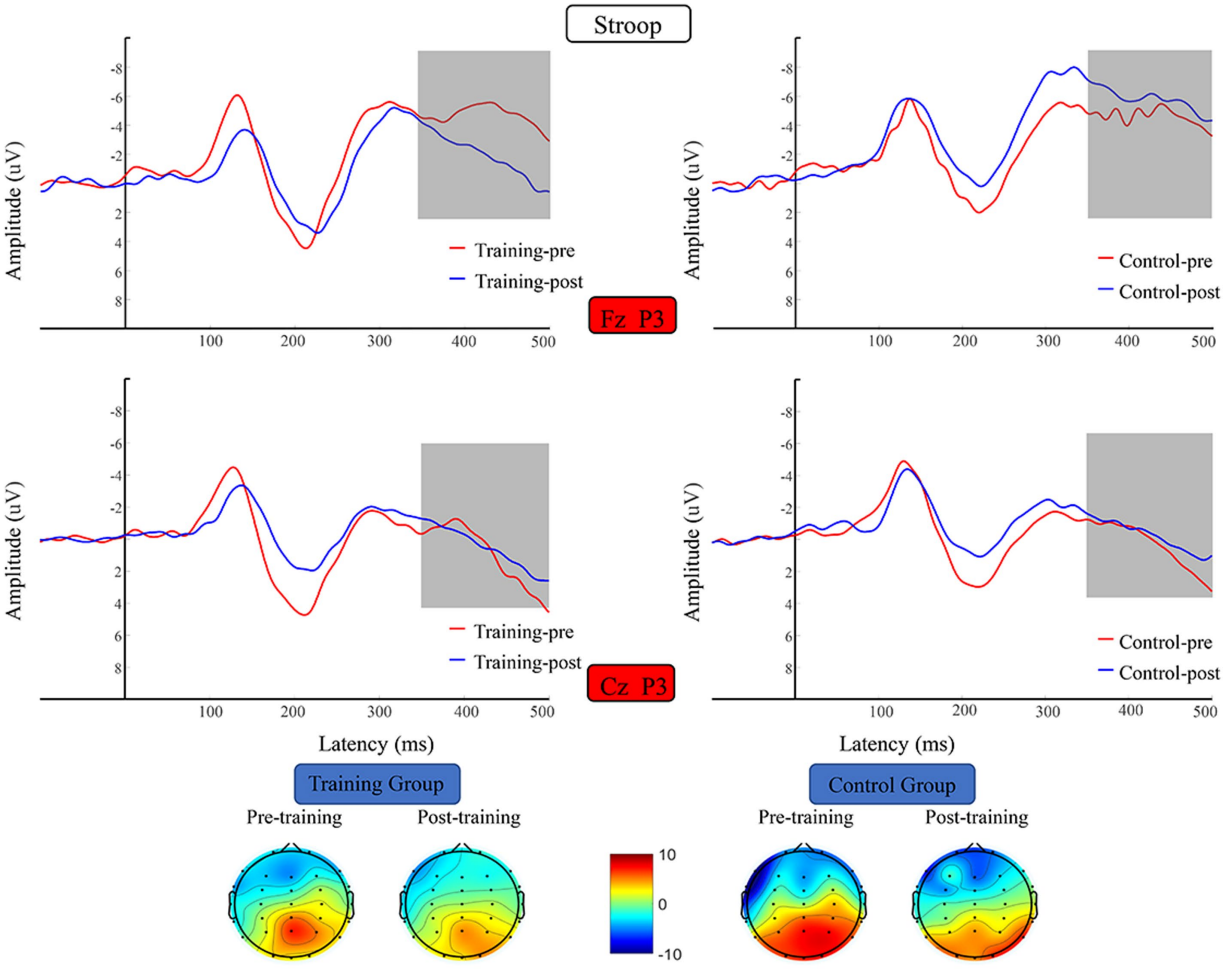
Waveforms and topographical maps of N2 for the two groups. Topographical maps were constructed using the mean amplitude in the N2 period (350–500 ms). Training-pre, training group pretest; Training-post, training group posttest; Control-pre, control group pretest; Control-post, control group posttest.

P3 average amplitude data were subjected to 2 (training group and control group) × 2 (pretest and posttest) × 2 (Fz, Cz) RM-ANOVA, which revealed the following. The main effect of time was significant (*F* (1, 38) = 29.259, *p* < 0.001, *η*^2^ = 0.435), and a significant channels × time × group interaction (*F* (1, 38) = 66.474, *p* = 0.031, *η*^2^ = 0.117), with no other significant. The data on the mean amplitude of P3 along the Fz and Cz channels were subjected to 2 (training group, control group) × 2 (pretest, posttest) RM- ANOVA, which did not reveal any significant difference in alpha energy before versus after the training period (Figure 9).

#### Time–frequency analysis

**Figure 10:**
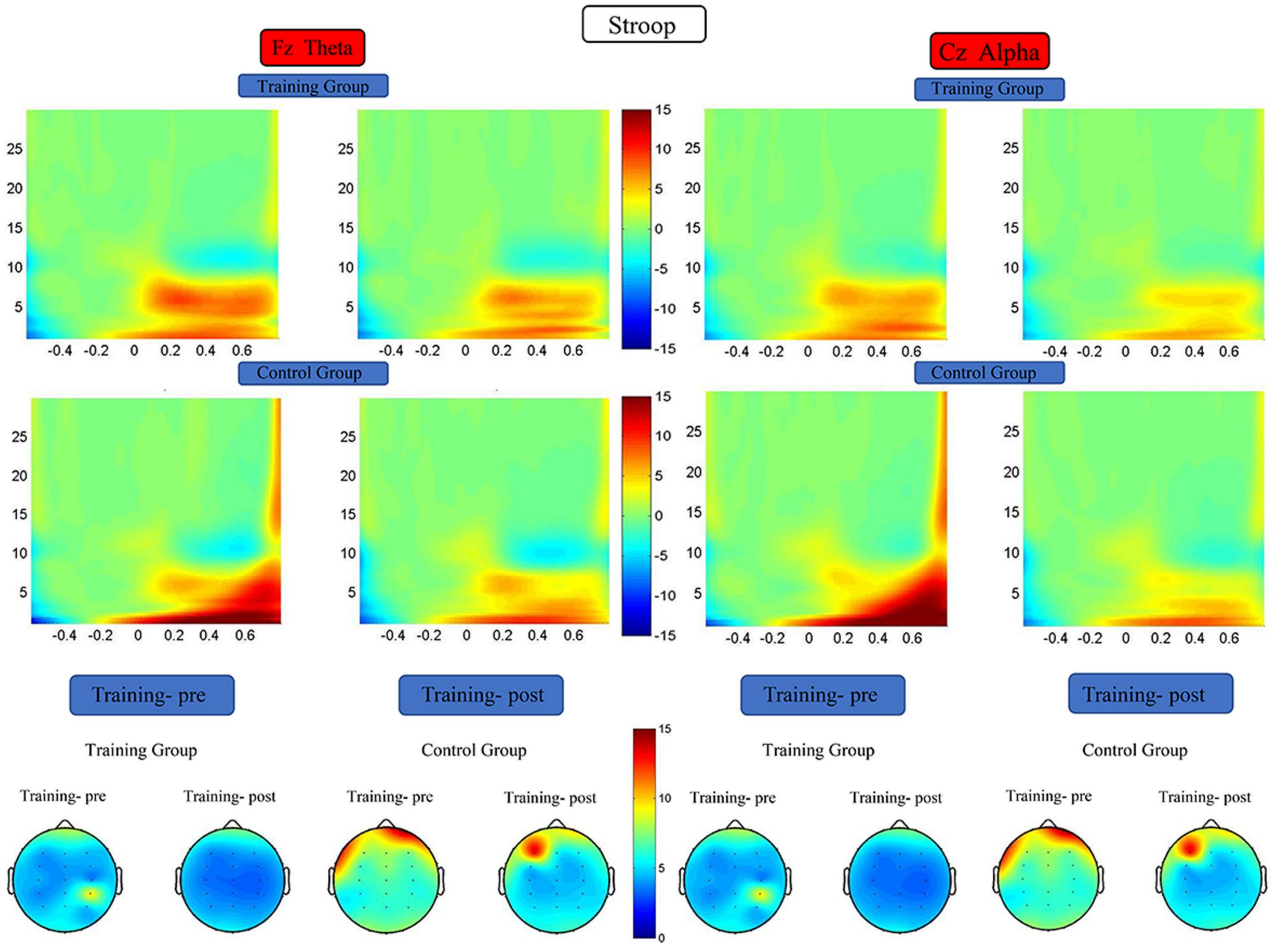
Spectrograms of mean ERSP of the two groups at electrode Fz and Cz. Training-pre, training group pretest; Training-post, training group posttest; Control-pre, control group pretest; Control-post, control group posttest.

The RM-ANOVA results did not indicate any significant difference in theta and alpha energy before versus after the training period (Figure 10).

### Pearson’s correlation

Pearson’s correlation analysis was performed for the index data for which significant changes were detected in the training group.

**Figure 11:**
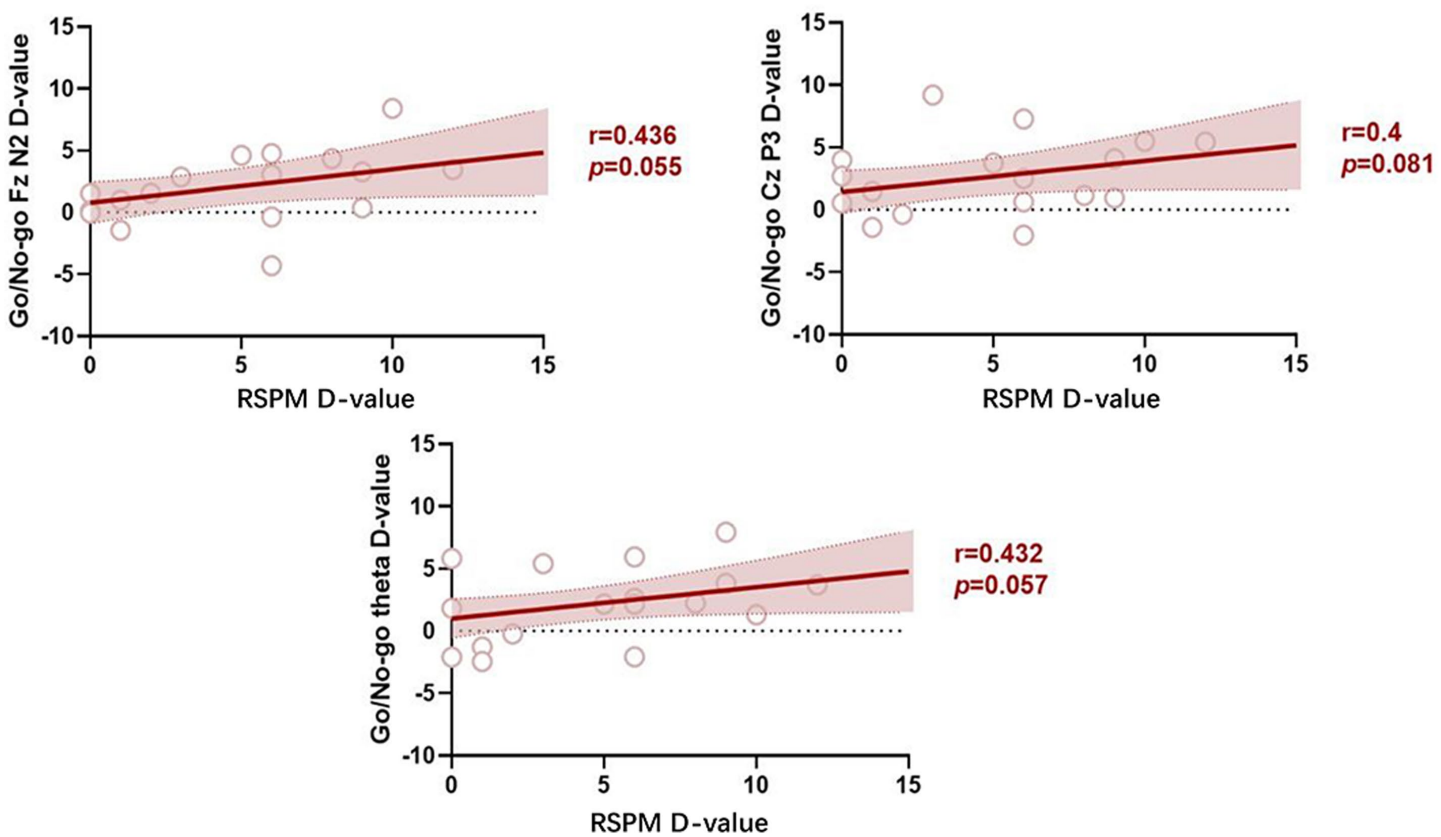
Correlations pertaining to differences in indicator values before versus after the training.

The results indicated that the change in fluid intelligence was positively correlated with the change in the mean amplitude of N2 along Fz (r = 0.436, *p* = 0.055), the mean amplitude of P3 along Cz (r = 0.400, *p* = 0.081), and the change in theta energy (r = 0.432, *p* = 0.057) for the go/no-go task (Figure 11).

## Discussion

In the present study, we investigated changes in inhibition control during far transfer of the effects of working memory training to fluid intelligence from an electrophysiological perspective. The results indicated that after 20 days of working memory training, the experimental group participants’ fluid intelligence performance and response inhibition were significantly improved. Specifically, the N2 and P3 average amplitude levels of the experimental group participants during no-go trials, which reflected their individual response inhibition, changed significantly. For the Stroop task, no significant change was detected in any indicator that reflected individual interference control. Contrary to our hypothesis, the individual theta band energy level was lower after the training. The Pearson’s correlation and regression analyses revealed that the improvement in response inhibition was a significant predictor of improved fluid intelligence.

Studies have demonstrated that the training effect of working memory can be far transferred to fluid intelligence and other cognitive abilities (Jaeggi et al., 2011). Zhao et al. (2011) reported that the fluid intelligence of children was significantly improved after they had received working memory training. This finding was then validated in children with learning disabilities, suggesting that this far-transfer effect is widespread (Chen et al., 2017). Our results verify this finding.

In the present study, the electrophysiological indexes of the participants’ response inhibition had changed significantly after they had received 20 days of working memory training. Specifically, this change manifested as a significant decrease in the amplitude of N2 during no-go trials. Rico-Picó et al. (2021) compared the N2 amplitudes of children with various levels of fluid intelligence and reported that the N2 amplitude level of children with high fluid intelligence was significantly lower than that of children with low fluid intelligence. This finding is consistent with the results of the present study. N2 is a key component of conflict monitoring and represents the level of involvement of an individual’s top-down response inhibition. Larger N2 amplitude indicates investment of more individual response inhibition resources. However, developmental studies have demonstrated that the amplitude of N2 decreases with age, which may be attributed to a gradual increase in an individual’s inhibition control efficiency with age (Hämmerer et al., 2010; Lo, 2018). The aforementioned findings indicate that working memory training increases the effectiveness of an individual’s conflict monitoring, and processing efficiency is a key factor influencing fluid intelligence (Hilger et al., 2017a).

The present study revealed that participants who received 20 days of working memory training exhibited significantly decreased P3 amplitude under no-go conditions when they were completing the response inhibition task. Several studies have asserted that P3 reflects individual response inhibition, whereas others have argued that P3 represents an overall evaluation of the inhibition control process (Righi et al., 2009; Wessel, 2018). The results of the present study support the first view, that is, the P3 component is more related to individual response inhibition than to overall inhibition control. This finding is consistent with those of most studies (Benikos et al., 2013; Wessel, 2018). The present study also discovered that the amplitude of P3 under no-go conditions was significantly higher after the training. Benikos et al. (2013) suggested that the P3 induced under no-go conditions is related to the selective attention and behavior selection process. Larger P3 amplitude after training indicates that individuals can better use inhibitory control resources to select behaviors during the response inhibition process.

In the present study, the theta band energy level of the participants was significantly lower after they had received the training. An increase in theta energy in the frontal lobe represents the level of top-down resource input that is involved during the process of identifying, monitoring, and resolving a problem as part of an inhibition control task (Nigbur et al., 2012; PastöTter et al., 2013; Cavanagh and Frank, 2014). In the present study, the theta energy level in the prefrontal cortex of the participants who received training was significantly decreased, suggesting an improvement in the efficiency of individual response inhibition. When the change in N2 components is also considered, the findings collectively suggest that the effect of working memory training on individual response inhibition is similar to the results of normal individual development and that working memory training further promotes development of individual response control, which is consistent with the neural efficiency hypothesis (Micheloyannis et al., 2006).

The results of the present study revealed that the participants’ alpha band energy levels were not significantly different after they had received the training. The idling hypothesis posits that a change in alpha band energy is related to brain excitability and represents an individual’s investment of attention resources in the current task and the degree of inhibitory control that they have over irrelevant brain regions (Pfurtscheller et al., 1996). However, the present study did not reveal any significant change in alpha band energy after the training, which may have been related to the excessively short reaction time window that was set in the experimental program.

Consistent with our hypothesis, the working memory training did not cause the participants to undergo a significant change for the Stroop task. This finding is consistent with those of previous research. In a study conducted by Zhao et al. (2018), participants received 14 days of working memory training, and the results indicated that the training effects did not transfer to the Stroop task. This finding was also supported by the results of subsequent studies (Zhao et al., 2020). Although the Stroop and go/no-go tasks both involve individual inhibition control, they are essentially different. Friedman and Miyake (2004) argued that interference control occurs at a later time point than response inhibition. To fully implement interference control, interference information must first be distinguished and this is then followed by a response. By contrast, the completion of response inhibition only requires the current reaction to be stopped in time, so smaller resources are required for inhibition control than for interference control. Therefore, from the perspective of improving go/no-go task indicators, working memory training improves the efficiency with which individuals use inhibition control resources, but this improvement can only be transferred to go/no-go tasks that require smaller cognitive resources. Nevertheless, other possible explanations cannot be ruled out. For example, the Stroop and go/no-go tasks involve different cognitive processes. From the perspective of attentional control, the Stroop task involves an inhibitory response to input stimuli, which emphasizes bottom-top cognitive processing. Conversely, the go/no-go task involves an individual’s inhibition of their own response output, which emphasizes top-down cognitive processing (Avital-Cohen and Tsal, 2016; Hong et al., 2017). Several studies have reported that, from the perspective of attention training, the efficiency of an individual’s attention network is considerably improved after they have received working memory training, and the activation mode is more mature (Rueda et al., 2005, 2012). This improvement may have a positive effect on top-down cognitive processing patterns, such that the training transfer effect for a go/no-go task is stronger.

Finally, Pearson’s correlation was performed in the present study to examine significant changes in index values after the training. The results indicated that improvement of response inhibition is a positive predictor of improved fluid intelligence. This finding suggests that the effect of working memory training on fluid intelligence can be achieved by enhancing response inhibition.

Various intelligence theories have posited that individual fluid intelligence is strongly correlated with inhibition control because of large overlapping networks in the brain (Rueda, 2018). Brain imaging studies have demonstrated that specific frontoparietal networks involved in inhibitory control are activated when individuals perform tasks requiring fluid intelligence, with these networks including the dorsolateral, ventrolateral prefrontal, and dorsal cingulate networks (Duncan et al., 2000; Duncan and Owen, 2000). This finding is consistent with the parietofrontal integration theory of intelligence. This theory holds that individual fluid intelligence is based on the information integration ability of the frontoparietal network, and information-processing efficiency is related to the level of individual fluid intelligence (Jung and Haier, 2007; Langer et al., 2012;Hilger et al., 2017b). From the perspective of individual brain development, the development trajectory of individual inhibition control ability overlaps extensively with that of fluid intelligence, and the frontoparietal network of children with higher fluid intelligence has a more efficient information-processing mode (Langeslag et al., 2013; Vendetti and Bunge, 2014; Fjell et al., 2015). Munakata et al. (2012) also argued that children who are more efficient at implementing internally driven (top-down) response inhibition are better at internalizing and manipulating abstract representations. Stimulus-driven (bottom-top) behavioral inhibition does not contribute to this phenomenon (Liu et al., 2022; Munakata et al., 2012). The results of the present study further validate the aforementioned concept, that is, the response inhibition of children can be increased through working memory training. This is a more mature response inhibition model that contributes to the improvement of fluid intelligence and is primarily based on reasoning.

Nevertheless, the present study has several limitations. For example, it demonstrated a significant positive correlation between fluid intelligence and response inhibition but did not clearly prove a causal relationship between these two factors. In addition, the small sample size and lack of an active control group also limit the generalizability of the results. However, the results partly reflect the role of response inhibition during transfer of the effects of working memory training to fluid intelligence. Future studies should explore the dynamic development of this transfer process, the duration of the transfer effect, and the factors that influence the transfer effect.

## Conclusion

The results of the present study suggest that working memory training can effectively improve the fluid intelligence of children, which is achieved by improving their individual response inhibition ability. This conclusion is that working memory training is an effective intervention method, which is of important implications to the intervention research on improving children’s fluid intelligence in the future. Inhibition control or attention control is one of the important factors of children’s fluid intelligence. Inhibitory control or attentional control ability is one of the important factors of fluid intelligence in children, and there is an interaction between individual intelligence system and inhibitory control system (Ye et al., 2017). This provides a theoretical basis for improving children’s fluid intelligence by intervention with the inhibition control ability of individuals. The intervention of fluid intelligence in the future should be explored from the aspect of individual response inhibition to further clarify its role in the development of individual fluid intelligence.

## Data availability statement

The raw data supporting the conclusions of this article will be made available by the authors, without undue reservation.

## Ethics statement

The studies involving human participants were reviewed and approved by the Ethics Committee of the Department of Psychology, Nanjing University. Written informed consent to participate in this study was provided by the participants’ legal guardian/next of kin.

## Author contributions

LW analyzed the data and drafted the manuscript. AS designed the study, recruited participants, and conducted training sessions. LC edited the manuscript. RZ analyzed the data and edited the manuscript. All authors contributed to the article and approved the submitted version.

## Funding

This work was supported by the Fundamental Research Funds for the Central Universities [2020300048] and Nanjing Institute of Minor Mental Health Research [2020ZK-ZK05].

## Conflict of interest

The authors declare that the research was conducted in the absence of any commercial or financial relationships that could be construed as a potential conflict of interest.

## Publisher’s note

All claims expressed in this article are solely those of the authors and do not necessarily represent those of their affiliated organizations, or those of the publisher, the editors and the reviewers. Any product that may be evaluated in this article, or claim that may be made by its manufacturer, is not guaranteed or endorsed by the publisher.
